# Dysrhythmia due to Verapamil Overdose in a Young Woman

**DOI:** 10.1155/cric/8747772

**Published:** 2026-02-19

**Authors:** Abdollah Malakian, Ensiyeh Taghizadeh, Zakaria Zakariaei

**Affiliations:** ^1^ Department of Emergency Medicine, Faculty of Medicine, Mazandaran University of Medical Sciences, Sari, Iran, mazums.ac.ir; ^2^ Department of Forensic Medicine and Toxicology, Mazandaran Registry Center for Opioid Poisoning, Antimicrobial Resistance Research Centers, Imam Khomeini Hospital, Mazandaran University of Medical Sciences, Sari, Iran, mazums.ac.ir

**Keywords:** dysrhythmia, intentional, overdose, verapamil

## Abstract

Verapamil (VER) is characterized as a calcium channel blocker (CCB) that can be intentionally or unintentionally overdosed. This drug, like other CCBs, inhibits the entry of calcium into vascular and myocardial tissues through L‐type channels. VER exerts negative inotropic and chronotropic effects on the myocardium through antagonistic L‐type calcium channels, and an overdose of this drug can result in a decrease in cardiac output, hypotension, and shock. In this report, we describe a unique case of VER toxicity in a 30‐year‐old woman who experienced a dysrhythmia and heart block after intentionally ingesting 800 mg of sustained‐release VER tablets.

## 1. Introduction

Calcium channel blockers (CCBs) are utilized to manage various medical conditions such as angina pectoris, hypertension, and cardiac arrhythmias. These drugs are available in both immediate‐release and sustained‐release formulations, with the latter being commonly used in therapeutic settings [1].

On the basis of their primary physiological effects, CCBs can be categorized into two groups: dihydropyridines, which selectively block L‐type calcium channels in the vasculature, and nondihydropyridines, such as diltiazem and verapamil (VER), which specifically target L‐type calcium channels in the myocardium [1, 2]. Although VER and diltiazem toxicity results in peripheral vasodilation, decreased cardiac inotropic, and bradycardia, dihydropyridine toxicity frequently leads to arterial vasodilation and reflex tachycardia [3]. The majority of CCBs possess a high distribution volume and a binding protein, and they undergo hepatic metabolism. As the dosage of CCBs increases, the rate of drug clearance slows down, leading to a prolonged half‐life. To facilitate once‐daily dosing, extended‐release formulations release the drug gradually from a matrix. This attribute renders the absorption uncertain in cases of overdose and prolongs the duration of toxicity [4].

A review of analytical methods for quantitative analysis of VER in pharmaceutical formulations and biological samples has revealed several techniques, including optical, chromatographic, electrochemical, conductometric, and other new methods. Of these, high‐performance liquid chromatography (HPLC) is the most commonly used method for VER analysis in blood samples. These analytical methods offer excellent sensitivity and robustness for VER measurement and are considered the methods of choice for therapeutic drug monitoring (TDM) of VER [5].

The therapeutic and toxic levels of VER can vary depending on the individual patient and the specific condition being treated. However, in general, the therapeutic range for VER in the blood is between 50 and 250 ng/mL. The toxic dose levels of VER are generally found in blood concentrations exceeding 500 ng/mL. Due to VER′s narrow therapeutic index, TDM is highly recommended to ensure optimal efficacy and minimize the risk of toxicity associated with VER therapy [6].

In instances of CCB poisoning, the most common manifestations are hypotension and bradycardia, especially in cases of VER or diltiazem overdoses. Nonetheless, even severe overdoses of dihydropyridines, such as nifedipine, have been associated with bradycardia. In some cases, heart failure symptoms like jugular venous distension and pulmonary crackles may be present. Due to the neuroprotective properties of CCBs, patients with CCB poisoning may remain lucid despite their hypotension. However, once cerebral perfusion is significantly reduced, the neurological state may abruptly deteriorate. Severe intoxication symptoms may be present in patients who consume 5–10 times the recommended dose or more [7].

Here, we describe a rare case of VER toxicity in a 30‐year‐old female patient who developed a cardiac arrhythmia and heart block after intentionally ingesting 800 mg of sustained‐release VER (20 tablets of 40 mg each). The patient′s condition improved with the aid of supportive therapy and temporary implantation of a cardiac pacemaker (TPM).

## 2. Case Presentation

**Figure 1:**
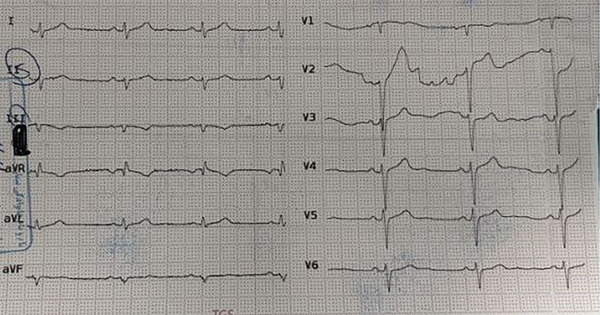
Initial ECG on admission.

**Figure 2:**
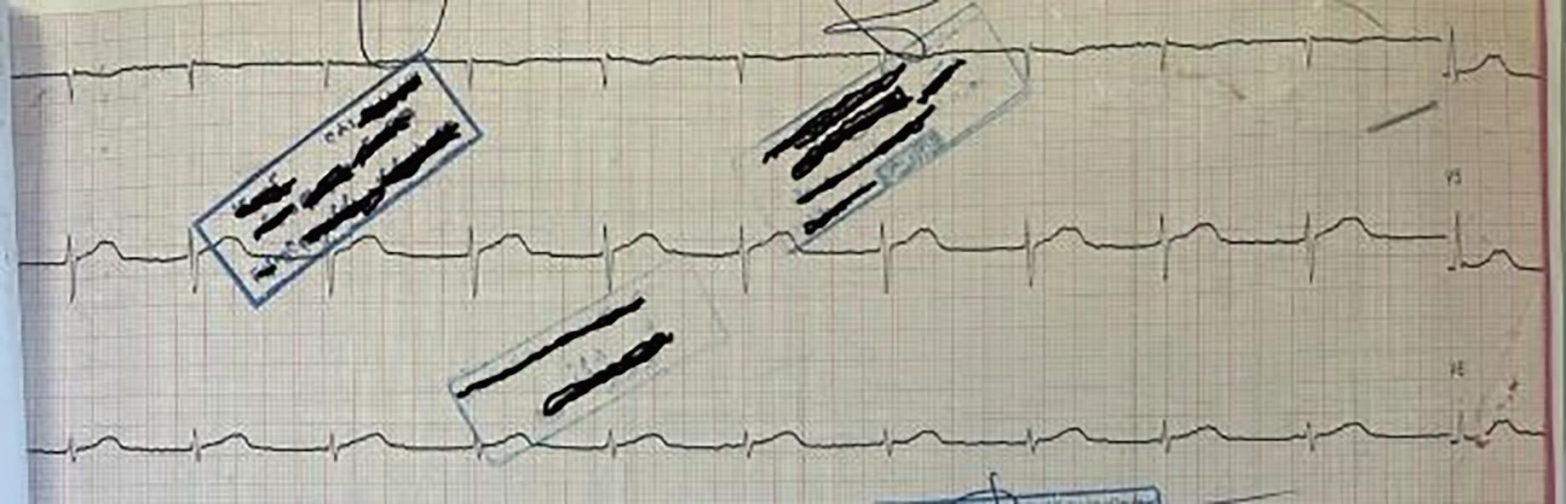
The cardiac junctional rhythm in electrocardiogram.

On August 15, 2022, a 30‐year‐old female patient with a history of suicide ingested a total of 800 mg of sustained‐release VER tablets (20 tablets of 40 mg each). She was admitted to the emergency room of a hospital in northern Iran approximately 6 h after the overdose. The patient presented with symptoms of weakness, lethargy, nausea and vomiting, and heart palpitations. On clinical examination, the patient was alert but uncooperative, presenting with a blood pressure of 90/65 mmHg, a heart rate of 60 beats per minute, a respiratory rate of 18 breaths per minute, and a body temperature of 36.8°C (Figure 1). Upon arrival at the emergency department, the patient was promptly administered serum dextrose saline at a rate of 20 mL per minute, along with atropine (1 mg) and norepinephrine at a rate of 5 *μ*g/min. Gastric lavage was performed after the insertion of a nasogastric tube, and 60 g of activated charcoal (AC) and sorbitol (40 mL) were administered. The patient was then transferred to the cardiac care unit (CCU). Initial electrocardiographic evaluation revealed a junctional rhythm (Figure 2).

Furthermore, the patient received intravenous (IV) calcium gluconate at a dose of 1 g of 10% solution every 8 h—directly antagonizing the toxicity by increasing extracellular calcium availability, thereby improving myocardial inotropy, vascular tone, and atrioventricular (AV) nodal conduction—along with glucagon (50 *μ*g/kg administered over 1 min, followed by an IV infusion of 1–5 mg/h), with the aim of enhancing perfusion and elevating the heart rate above 50 beats per minute (glucagon activates adenylate cyclase independently of beta‐receptors, thereby elevating cyclic adenosine monophosphate [cAMP] levels to augment cardiac contractility and heart rate). High‐dose insulin euglycemic therapy (HIET) was initiated to enhance myocardial contraction, consisting of a regular insulin bolus of 60 units (1 unit/kg) followed by an infusion at 30 units per hour (0.5 unit/kg/h), along with dextrose 50% administered at 60 g per hour (HIET improves myocardial contractility in VER toxicity by enhancing cardiac myocyte glucose uptake and metabolism, thereby countering the drug′s impairment of energy substrate utilization).

**Figure 3:**
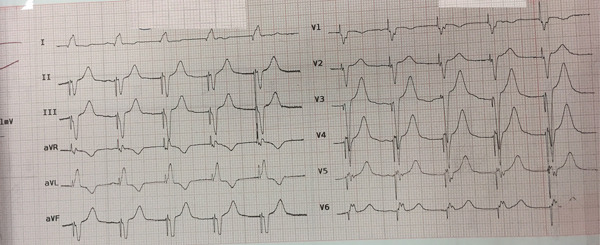
Ventricular paced rhythm.

**Figure 4:**
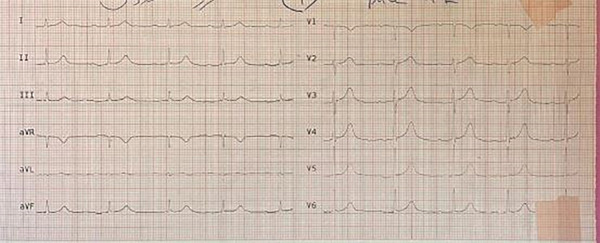
Normal sinus rhythm after recovery.

The laboratory blood test results, which are presented in Table 1 and were obtained during the patient′s hospitalization, indicate a gradual improvement of hyperglycemia, metabolic acidosis, and the increase of liver enzymes with appropriate treatment and hydration in the subsequent days. Subsequently, 6 h following admission to the CCU ward, the patient exhibited symptoms of drowsiness, hypotension, dysrhythmia, and heart block. The patient was promptly transferred to the catheterization and interventional cardiology unit, where TPM implantation successfully restored a ventricular paced rhythm (Figure 3). After 48 h, the patient′s vital signs stabilized and sinus rhythm was restored, permitting the initiation of oral intake (Figure 4). Psychotherapy sessions commenced 72 h after admission. One week later, the patient was discharged from the hospital in good condition with a recommendation for continued psychotherapy at a psychiatric clinic. Written informed consent was obtained from the patient′s legally authorized representative to publish this report in accordance with the journal′s patient consent policy. This study was conducted according to the Declaration of Helsinki Principles. Also, CARE guidelines and methodology have been followed in this study.

**Table 1:** Baseline laboratory results.

Parameter	First day	Second day	Third day	Reference value
BUN	15.5	16	11	9–26 mg/dl
Cr	1.54	1.19	0.9	0.6–1.2 mg/dl
BS	234	68	130	? 200 mg/dl
Na	143	146	141	136–145 mEq/L
K	3.9	5	3.9	3.5–5.1 mEq/L
Mg	2.25	2.14	1.66	1.9–2.5 mg/dl
Ca	8.5	8.2	8.5	8.5–10.5 mg/dl
P	2.8	5.9	3.1	2.6–4.5 mg/dl
LDH	328	—	—	< 480 U/L
CRP	2	—	—	Up to 6 mg/l
AST	910	383	25	10–40 U/L
ALT	1210	918	13	? 45 U/L
ALP	95	134	136	64–306 U/L
Alb	4.3	4.3	—	3.5–5 g/dl
Troponin	10 ng/dl	—	—	< 100 ng/dl
TG	—	51	—	< 200 mg/dl
CHOL	—	72	—	< 200 mg/dl
PT	16.3	—	—	11–13 Sec
PTT	27	—	—	19–36 Sec
INR	1.34	—	—	1–1.3 Ratio
PH	7.24	7.28	7.37	7.35–7.45
PCO_2_	39	29	30	35–45 mmHg
HCO_3_	16.7	13.6	17.8	22–28 mEq/L
Lactate	—	3.3	2.1	1.4–2.3 mmol/L
HGB	12.9	—	14	11.5–15.5 g/dl
RBC	4.61	—	4.78	4.50–5.30 × 10^6^
WBC	15200	—	11400	4000–10000/mm^3^
PLT	315000	—	207000	150–450 × 10^3^/mm^3^

Abbreviations: Alb, albumin; ALT, alanine aminotransferase; ALP, alkaline phosphatase; AST, aspartate aminotransferase; BS, blood sugar; BUN, blood urea nitrogen; ca, calcium; CHOL, cholesterol; Cr, creatinine; CRP, C‐reactive protein; HCO_3_, bicarbonate; HGB, hemoglobin; INR, international normalized ratio; K, potassium; LDH, lactate dehydrogenase; Mg, magnesium; Na, sodium; P, phosphorus; PCO_2_, pressure of carbon dioxide; pH, potential of hydrogen; PLT, platelet; PT, prothrombin time; PTT, partial thromboplastin time; RBC, red blood cell; TG, triglycerides; and WBC, white blood cell.

## 3. Discussion

In CCB poisoning, inhibition of calcium‐mediated insulin release may lead to hyperglycemia, though this increase is typically not of significant clinical consequence beyond diagnostic considerations. Hyperglycemia may be a useful indicator for differentiating between CCB and beta‐blocker poisoning in nondiabetic patients. Bradydysrhythmia and PR interval prolongation are electrocardiogram abnormalities commonly associated with CCB overdose [8]. Over the past several years, the use of sustained‐release VER has been associated with an uptick in reported cases of VER toxicity in the medical literature. As a result, the most frequently observed and serious adverse effects, such as bradycardia, hypotension, and abnormal conduction, have been widely documented (with complete AV block being the most severe potential consequence) [9].

The metabolic disturbances that follow VER toxicity may include hyperglycemia resulting from decreased insulin release, metabolic acidosis due to increased lactate generation in response to hypoperfusion, and imbalances in blood potassium levels. In rare cases, additional complications such as stroke have also been reported [10]. The severity of these adverse effects can result in fatal overdoses, which have been documented with VER at doses ranging from 800 mg to 24,000 mg [11]. However, determining an accurate ingested amount can be challenging in cases of overdose. Our case involved the intentional ingestion of 800 mg of sustained‐release VER tablets, which resulted in a significant elevation in blood lactate levels (3.3 mmol/L), experienced drowsiness, and metabolic acidosis.

Victims of sustained‐release VER toxicity will eventually experience the same adverse effects as those using standard formulations, but symptoms may appear later. Standard VER toxicity typically presents within 2–4 h, whereas toxicity from sustained‐release formulations may take up to 12 h to manifest [7]. Additionally, signs and symptoms of sustained‐release VER poisoning may persist for 48–72 h, likely due to the drug′s prolonged half‐life, delayed absorption in the gastrointestinal tract, or saturation of VER clearance pathways. In cases of significant overdose, sustained‐release VER deposits have been identified in the GI tract, occasionally leading to the formation of pharmacobezoars [12].

The primary goals of treatment for CCB poisoning are initial resuscitation and hemodynamic stabilization. Despite appropriate treatment, bradycardia and hypotension may persist and be difficult to manage. IV fluids are the first‐line treatment for hypotension, whereas atropine is the first‐line treatment for bradycardia. In cases of potentially dangerous ingestion (greater than 5–10 times the standard dose) presenting within 1–2 h, insertion of a nasogastric tube and orogastric lavage may be necessary [13]. AC should be administered to patients with CCB overdoses, even if they are asymptomatic. It is most effective when given within an hour of ingestion, but it may be effective for individuals who experience delayed effects from CCBs. Whole bowel irrigation (WBI) may be useful in cases where the patient has ingested a sustained‐release or extended‐release preparation, such as VER or diltiazem [14]. In this case study, TPM placement may be necessary for symptomatic bradycardia or dysrhythmia and heart block despite initial resuscitation and hemodynamic stabilization with IV fluids, vasopressors, calcium gluconate, glucagon, and HIET.

St‐Onge in a systematic review showed that in severe CCB poisonings, if there are signs of severe hypotension and/or bradycardia, clinicians should consider the following treatments: airway stabilization as needed; IV boluses of isotonic crystalloid, calcium gluconate, and glucagon; HIET; vasopressors; and IV lipid emulsion therapy [15]. The use of a pacemaker, which is recommended for significant bradycardia or high‐grade conduction blocks, can also be effective in individuals with CCB overdose [16].

A limitation of our study is the unavailability of VER blood level measurements, as this test is not routinely performed in the hospitals affiliated with Mazandaran University of Medical Sciences, Sari, Iran.

## 4. Conclusion

Because very low doses of VER can cause lethality, CCB toxicity may not produce significant consciousness disorders despite the presence of hypotension and bradycardia. However, in the treatment of VER poisoning, it is necessary to pay attention to the following: airway stabilization, IV boluses of isotonic crystalloid, AC, WBI in cases of pharmacobezoars formation, and administration of drugs such as vasopressors, calcium gluconate, glucagon, HIET, and finally; the basis for TPM placement is symptomatic bradycardia or dysrhythmia and heart block.

## Author Contributions

A.M. and E.T. are involved in the collecting of samples and data. Z.Z. participated in the interpretation, writing, editing of the manuscript. Z.Z. prepared the draft and submitted the manuscript.

## Funding

This work was supported by the Mazandaran University of Medical Sciences, 10.13039/501100004160.

## Disclosure

All authors reviewed and approved the final version of the manuscript.

## Ethics Statement

This research was approved by the Mazandaran University of Medical Science Ethics Committee (No: IR.MAZUMS.REC. 1403.554) and was carried out in accordance with the Helsinki Declaration Principles.

## Consent

Written informed consent was obtained from the patient′s legally authorized representative to publish this report in accordance with the journal′s patient consent policy.

## Conflicts of Interest

The authors declare no conflicts of interest.

## Data Availability

The data that support the findings of this study are available from the corresponding author upon reasonable request.
